# Multiple drug resistance-associated protein (MRP4) exports prostaglandin E2 (PGE2) and contributes to metastasis in basal/triple negative breast cancer

**DOI:** 10.18632/oncotarget.14145

**Published:** 2016-12-24

**Authors:** Tyler J Kochel, Jocelyn C Reader, Xinrong Ma, Namita Kundu, Amy M Fulton

**Affiliations:** ^1^ University of Maryland School of Medicine, Department of Pathology, Baltimore, MD, USA; ^2^ University of Maryland School of Medicine, Department of Obstetrics, Gynecology, and Reproductive Sciences, Baltimore, MD, USA; ^3^ Marlene and Stewart Greenebaum Comprehensive Cancer Center, Baltimore, MD, USA; ^4^ Baltimore Veterans Affairs Medical Center, Baltimore, MD, USA

**Keywords:** breast cancer, metastasis, MRP4, PGE_2_, TNBC

## Abstract

Cyclooxygenase-2 (COX-2) and its primary enzymatic product, prostaglandin E2 (PGE2), are associated with a poor prognosis in breast cancer. In order to elucidate the factors contributing to intratumoral PGE_2_ levels, we evaluated the expression of COX-2/PGE_2_ pathway members MRP4, the prostaglandin transporter PGT, 15-PGDH (PGE_2_ metabolism), the prostaglandin E receptor EP4, COX-1, and COX-2 in normal, luminal, and basal breast cancer cell lines. The pattern of protein expression varied by cell line reflecting breast cancer heterogeneity. Overall, basal cell lines expressed higher COX-2, higher MRP4, lower PGT, and lower 15-PGDH than luminal cell lines resulting in higher PGE_2_ in the extracellular environment. Genetic or pharmacologic suppression of MRP4 expression or activity in basal cell lines led to less extracellular PGE_2_. The key finding is that xenografts derived from a basal breast cancer cell line with stably suppressed MRP4 expression showed a marked decrease in spontaneous metastasis compared to cells with unaltered MRP4 expression. Growth properties of primary tumors were not altered by MRP4 manipulation. In addition to the well-established role of high COX-2 in promoting metastasis, these data identify an additional mechanism to achieve high PGE_2_ in the tumor microenvironment; high MRP4, low PGT, and low 15-PGDH. MRP4 should be examined further as a potential therapeutic target in basal breast cancer.

## INTRODUCTION

Breast cancer is the most frequently diagnosed cancer among women in the U.S. and is the second leading cause of cancer-related mortality in this population [1]. Metastatic disease is the cause of the majority of cancer-related death [1]. Elucidating and targeting the processes that lead to metastasis could prevent mortality due to breast and other cancers. Breast cancer is a heterogeneous disease that is typically classified according to the expression of three cell surface receptors, i.e. estrogen receptor, progesterone receptor, and HER2 receptor [2]. Estrogen receptor and HER2 can be targeted therapeutically which leads to a more favorable prognosis for women with tumors expressing these receptors. Alternatively, 10-15% of all breast cancers do not express any of these receptors and are referred to as Triple Negative Breast Cancer (TNBC) [3]. Lacking tumor-specific molecular targets, TNBCs are treated via surgery, chemotherapy, and radiation but nevertheless have a worse prognosis compared to other subtypes of breast cancer [4].

Cyclooxygenase enzymes (COX-1 and COX-2) catalyze the rate-limiting step in the production of eicosanoids from arachidonic acid, an omega-6 fatty acid found in the cell membrane. The main eicosanoid product found in tumors is the inflammatory mediator prostaglandin E_2_ (PGE_2_) [5, 6]. Like other cancers of epithelial origin, aberrant expression of COX-2 is found in approximately half of all breast cancers, and elevated COX-2 and PGE_2_ levels are associated with a poor prognosis [5–10]. PGE_2_ is actively exported by multiple drug resistance-associated protein 4 (MRP4) into the extracellular space where it can bind one of four cognate, G-protein coupled receptors (EP1-4) and induce several signaling pathways [11–23]. Activation of EP2 and EP4 receptors has been associated with several contributing factors in tumor progression including angiogenesis, cellular proliferation, migration, invasion, metastasis, immune evasion, and support of a cancer stem-like cell phenotype [14–23]. MRP4 has been associated with a poor prognosis in tumors of the blood, brain, colon, liver, lung, pancreas, and prostate, but has only been briefly described in breast cancer [24–31]. Extracellular PGE_2_ can be imported through the prostaglandin transporter (PGT) via exchange with intracellular lactate and metabolized by 15-prostaglandin dehydrogenase (15-PGDH) [32–36]. 15-PGDH is a tumor suppressor gene in breast cancer as lack of sufficient expression of this enzyme can result in the accumulation of PGE_2_ thus leading to sustained PGE_2_ signaling [35, 37]. Metabolized PGE_2_ cannot bind EP receptors; therefore, PGT and 15-PGDH are both required to terminate PGE_2_-activated signaling [38].

Using large publicly available data sets, we recently reported that MRP4, PGT and 15-PGDH were differentially expressed among distinct breast cancer molecular subtypes [39]. In basal type breast cancer or TNBC, high COX-2, high MRP4, low PGT, and low 15-PGDH mRNA expression levels were observed. The current study examines the functional significance of this observation.

## RESULTS

**Figure 1:**
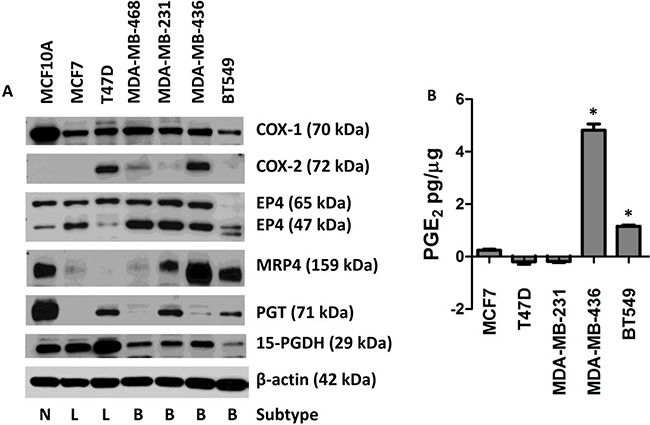
Expression of the COX-2/PGE2 pathway proteins in seven human breast cell lines leading to extracellular accumulation of PGE2 MCF10A cells were compared to six human breast cancer cell lines. **A**. Protein lysates from MCF10A, MCF7, T47D, MDA-MB-468, MDA-MB-231, MDA-MB-436, and BT549 cells were blotted for the following proteins: COX-1, COX-2, EP4, MRP4, PGT, and 15-PGDH. β-actin was used as a loading control. Breast cancer subtypes are indicated below the blot by Normal (N), Luminal (L), and Basal (B). **B**. Conditioned media was collected from sub-confluent cell culture and assayed for PGE_2_. Total PGE_2_ (pg) in the conditioned media was normalized to total cellular protein (μg). * p < 0.01 relative to the other four cell lines. PGE_2_ content is expressed as mean ± SEM of triplicate determinations.

Based on our previous observations that primary breast cancers of molecularly defined subtypes differentially expressed members of the COX-2/PGE_2_ pathway at the mRNA level, we characterized a panel of breast cancer cell lines reflecting breast cancer subtypes to further test the hypothesis that aggressive, metastatic, and basal type breast cancers would display a pattern of COX-2 pathway expression leading to high PGE_2_ in the tumor microenvironment. MRP4 protein expression was elevated in the MCF10A (normal mammary epithelium), MDA-MB-231 (basal), MDA-MB-436 (basal), and BT549 (basal) cell lines compared to MCF7 (luminal) and MDA-MB-468 (basal) cells (Figure 1A). MRP4 was not detected in T47D (luminal) cells. Overall, elevated MRP4 expression was somewhat correlated with cell lines of aggressive molecular subtype (basal) compared to luminal malignant cell lines.

MCF10A cells had the highest PGT protein expression (Figure 1A). T47D, MDA-MB-231, and BT549 cell lines displayed moderate levels of PGT while MCF7, MDA-MB-468, and MDA-MB-436 cell lines expressed either low or no detectable PGT. Expression of the PGE_2_-metabolizing enzyme, 15-PGDH, in MCF10A, MCF7, and T47D cells was higher than in the four basal cell lines (Figure 1A). The observed protein expression pattern for 15-PGDH is characteristic of a tumor suppressor gene [35, 37]. Consistent with a house-keeping role, COX-1 protein expression was detected in all cell lines (Figure 1A). COX-2 protein was detected in four of seven cell lines (Figure 1A).

The EP4 receptor has been detected at multiple sizes due to the extent of glycosylation of the protein; the apparent size ranging between 47 and 65 kDa. EP4 expression at the larger (65 kDa) isoform was detected at similar levels in all cell lines except BT549 which had barely detectable EP4 expression at this size. Higher expression of the 47 kDa isoform was detected in the basal cell lines compared to normal (MCF10A) and luminal cell lines (Figure 1A).

Taken together these studies reveal complex expression patterns of PGE_2_ family members in individual cell lines reflecting the marked heterogeneity of human breast cancer. In each cell line, a different combination of these proteins could lead to dysregulated levels of PGE_2_ in the tumor microenvironment; however, some general patterns have emerged. We saw that more aggressive cell lines generally express higher levels of MRP4 and reduced levels of PGT and 15-PGDH. The net result would be higher levels of PGE_2_ in the tumor microenvironment due to increased PGE_2_ export by MRP4 and decreased PGE_2_ import and metabolism via PGT and 15-PGDH, respectively. Conversely, less aggressive, luminal cell lines tended to have lower levels of MRP4, higher levels of PGT, and higher levels of 15-PGDH expression. These patterns are consistent with our previous report that primary basal type or TNBC breast cancers typically express high COX-2, high MRP4, low PGT, and low 15-PGDH mRNA, and also with our mRNA expression analysis of these cell lines (data not shown) [39].

Given these differential expression patterns of the PGE_2_ pathway members, we asked what impact these differences have on net PGE_2_ production by breast cancer cells. We analyzed PGE_2_ production in conditioned media via ELISA and saw that MDA-MB-436 cells produce high levels of PGE_2_ (4.82 pg/μg cellular protein) (Figure 1B). These cells express both COX-1 and COX-2 proteins along with elevated MRP4 and reduced PGT (Figure 1A). BT549 cells accumulate moderate levels of PGE_2_ (1.16 pg/μg cellular protein) in conditioned media (Figure 1B). These cells do not express high levels of COX-2; however, they express elevated levels of MRP4 while not expressing 15-PGDH that would metabolize any PGE_2_ produced from COX-1 (Figure 1A). PGT expression is also low in these cells so that extracellular PGE_2_ would not be imported efficiently and could accumulate in the conditioned media. PGE_2_ levels were low in the conditioned media of MCF7 cells (Figure 1B) consistent with the negligible expression of COX-2 and MRP4. Thus, PGE_2_ detected in the conditioned media would be dependent on COX-1 and export would be dependent on the low level of MRP4 or passive diffusion (Figure 1A). In addition, MCF7 cells express 15-PGDH which would suppress the amount of PGE_2_ detected in the conditioned media from these cells. T47D cells favor PGE_2_ metabolism as there was less PGE_2_ detected in the conditioned media compared to the unconditioned media (Figure 1B). The PGE_2_ detected in the unconditioned media likely came from the serum component of the growth media. Since T47D cells express PGT and high levels of 15-PGDH, they are poised to robustly metabolize PGE_2_, even though COX-2 is expressed (Figure 1A). These cells also lack MRP4 expression so PGE_2_ is not actively exported (Figure 1A). Likewise, a net decrease in PGE_2_ was also detected from the growth media of MDA-MB-231 cells (Figure 1B). This is consistent with the low endogenous COX-2 levels in these cells and moderate expression of MRP4, PGT, and 15-PGDH (Figure 1A). 15-PGDH expression in MDA-MB-436 cells is equal to other cells that do not accumulate PGE_2_ in the conditioned media, but since the ratio of MRP4-to-PGT is high, any PGE_2_ in the cells would be exported instead of being metabolized.

**Figure 2:**
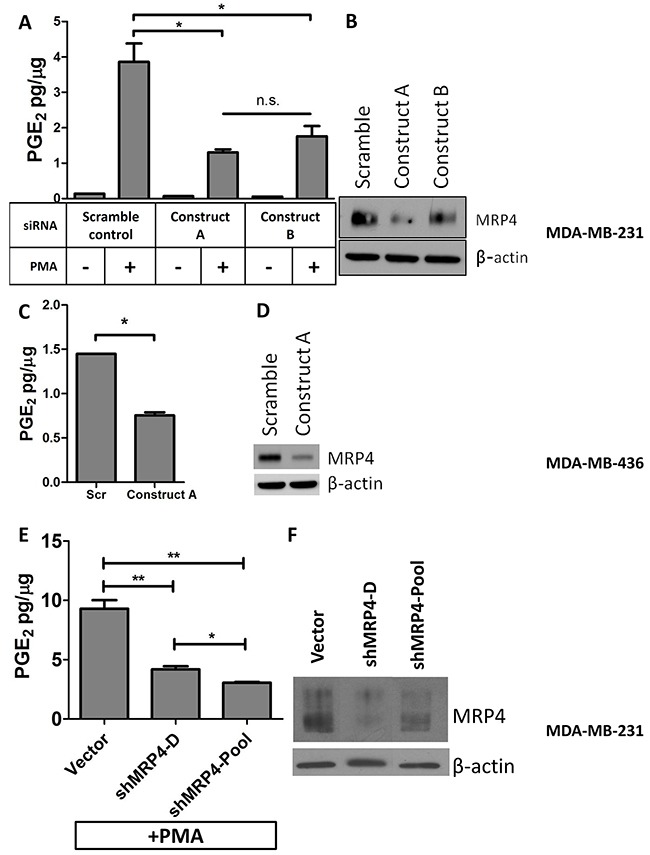
Knockdown of MRP4 in MDA-MB-231 and MDA-MB-436 cells suppresses the export of PGE2 MDA-MB-231 cells were transfected with MRP4 siRNA (3 nmol/L) for 24 hours before being stimulated with PMA (80 nmol/L, 1 hr) and replacing growth media. After 16 hours, conditioned media and total protein lysate was collected. **A**. Conditioned media from MRP4-silenced cells (MDA-MB-231) stimulated with PMA was assayed for PGE_2_ and total protein. **B**. A representative western blot of MDA-MB-231 cells transfected with siRNA (3 nmol/L) and stimulated with PMA shows decreased expression of MRP4. MDA-MB-436 cells were transfected with MRP4 siRNA (10 nmol/L). **C**. Conditioned media from MDA-MB-436 cells was collected after overnight incubation and assayed for PGE_2_ content. **D**. A representative western blot showing 68% decreased MRP4 expression following siRNA transfection of MDA-MB-436 cells. **E**. MDA-MB-231 vector control, clone (shMRP4-D), and pool (shMRP4-Pool) populations of stable MRP4 knockdown cells were briefly stimulated with 80 nM PMA and incubated overnight in fresh growth medium. Conditioned media was collected and assayed for PGE_2_. **F**. A representative western blot showing decreased MRP4 expression of MDA-MB-231 clone shMRP4-D and shMRP4-Pool cell lines compared to vector control cells. PGE_2_ is expressed as mean ± SEM pg/μg protein from triplicate determinations. β-actin was used as a loading control.* p < 0.05, ** p < 0.01, n.s. = not significant.

Since MRP4 exports PGE_2_ from a variety of cell types, elevated MRP4 expression in the tumor could be a mechanism by which malignant cells maintain elevated PGE_2_ in the tumor microenvironment [13, 25, 29–31, 40, 41]. In order to determine the role of MRP4 in PGE_2_ export from tumor cells, we used both genetic and pharmacologic approaches to perturb MRP4 activity and measured PGE_2_ accumulation in the conditioned media of two basal type cell lines. The MDA-MB-231 cell line expresses elevated levels of MRP4, and PGE_2_ production is inducible by inflammatory stimuli. The migratory and metastatic behaviors of MDA-MB-231 cells are partially dependent on PGE_2_ signaling [42]. The MDA-MB-436 cell line expresses high MRP4 and produces PGE_2_ without exogenous stimulation. MDA-MB-231 cells were treated with PMA (phorbol ester) to induce COX-2 and accumulation of PGE_2_ in the conditioned media [43]. We employed siRNA targeting of ABCC4 to reduce expression of MRP4 and examined the level of PGE_2_ in the conditioned media. Using two siRNA constructs, we achieved significant (35-75%) reduction in MRP4 expression relative to scramble control cells (Figure 2B). In MDA-MB-231 cells, stimulation with PMA (80 nM, 1 hr) resulted in a 25-fold increase in PGE_2_ accumulation in the conditioned media compared to unstimulated cells (Figure 2A). Fifty-four to sixty-six percent less PGE_2_ was exported from MDA-MB-231 cells when they were transfected with siRNA targeting ABCC4 and briefly stimulated with PMA compared to PMA-treated scramble siRNA control (p < 0.05). The PGE_2_ export role of MRP4 was confirmed in a second basal type cell line. Like MDA-MB-231 cells, MDA-MB-436 cells accumulated significantly less PGE_2_ in the conditioned media when MRP4 expression was reduced with siRNA (Figure 2C, 2D).

Stable MRP4 knockdown clones from MDA-MB-231 cells were also generated and characterized for decreased MRP4 expression. MRP4 protein expression was reduced by 62-67% relative to vector control (Figure 2E). MDA-MB-231 cells (one clone and one pooled population) with decreased MRP4 expression were briefly stimulated with PMA to induce COX-2 and to produce PGE_2_. As observed in transient MRP4 knockdown, cells with decreased MRP4 expression exported less PGE_2_ compared to vector control confirming the results obtained using transient gene silencing (Figure 2F).

**Figure 3:**
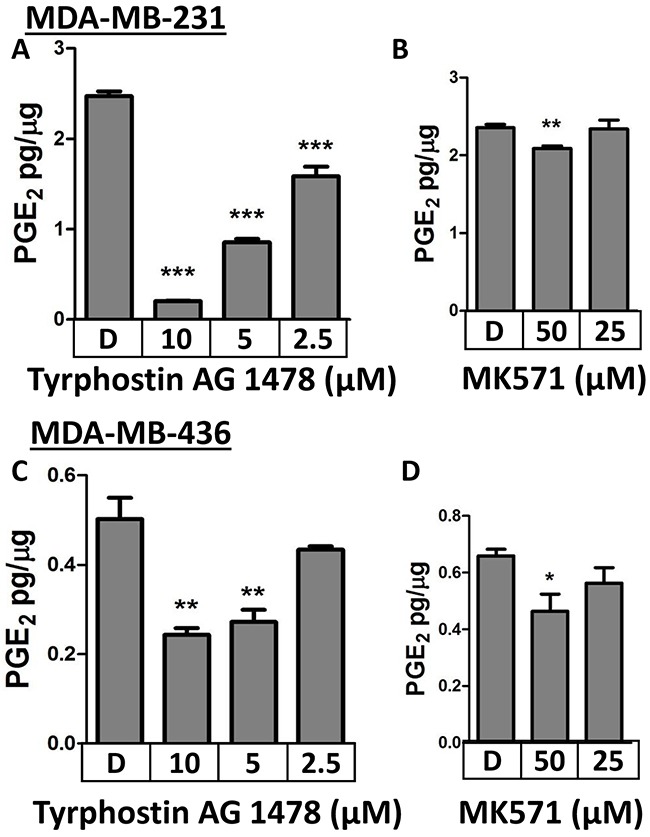
Pharmacologic inhibition of MRP4 with Tyrphostin AG1478 or MK571 suppresses PGE2 export **A**. MDA-MB-231 cells were stimulated briefly with 80 nM PMA before the media was replaced with the indicated concentrations of **A**. Tyrphostin, **B**. MK571, or DMSO. MDA-MB-436 cells were treated with the indicated concentrations of **C**. Tyrphostin **D**. MK571, or DMSO. PGE_2_ accumulation in the conditioned media after 18 hours was quantified by enzyme immunoassay. PGE_2_ is expressed as mean ± SEM pg/μg protein from triplicate determinations. * p < 0.05, ** p < 0.01*** p < 0.001 relative to DMSO.

Two pharmacologic inhibitors of MRP4 (Tyrphostin AG1478, MK571) were also employed to evaluate the role of MRP4 in PGE_2_ export. PMA-stimulated MDA-MB-231 cells or MDA-MB-436 cells were treated with inhibitor and evaluated for PGE_2_ accumulation in the conditioned media. Tyrphostin AG1478, a tyrosine kinase inhibitor with recently identified inhibitory activity on MRP4 [44], inhibited PGE_2_ accumulation in both cell lines in a dose-dependent manner (Figure 3A, 3C). In MDA-MB-231 cells, PGE_2_ export was decreased by 92%, 65%, and 36% in the presence of Tyrphostin AG1478 at 10, 5, and 2.5 μmol/L, respectively (p < 0.001). Likewise, PGE_2_ export by MDA-MB-436 cells was significantly decreased by 52%, 46%, and 14% in the presence of Tyrphostin AG1478 at 10, 5, and 2.5 μmol/L, respectively (p < 0.01). Treatment with 50 μM, but not 25 μM, MK571 resulted in a reduction in the level of PGE_2_ in the conditioned media of both MDA-MB-231 and MDA-MB-436 cells when compared to vehicle control (Figure 3B, 3D).

**Figure 4:**
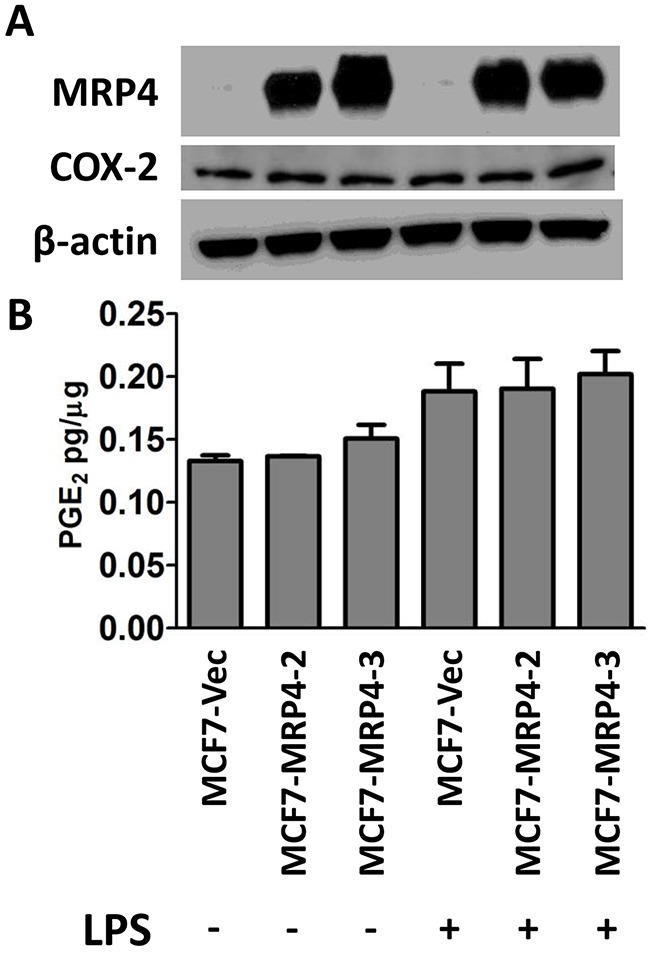
Stable over-expression of MRP4 does not enhance PGE2 export from MCF7 cells **A**. Vector-expressing control cells (MCF7-Vec) or MRP4-expressing cells (MCF7-MRP4-2, MCF7-MRP4-3) were stimulated with 10 μg/mL LPS for 24 hours. Total protein lysate was collected for western blot to determine relative levels of MRP4 and COX-2 expression. β-actin was used as a loading control. **B**. Conditioned media was collected and assayed for PGE_2_. PGE_2_ is expressed as mean ± SEM pg/μg protein from triplicate determinations. Western blot lanes are vertically aligned with corresponding PGE_2_ levels.

Suppression or inhibition of MRP4 decreases the export of PGE_2_ from basal or TNBC breast cancer cells. Conversely, MCF7 cells were used for MRP4 over-expression experiments due to the low level of endogenous MRP4 expression as well as being representative of an estrogen and progesterone receptor positive, luminal subtype cell line. MCF7 cells were transfected to express either empty vector control or an MRP4 expression plasmid (pcDNA3.1(-)-MRP4-Zeo) and two lines were selected (MCF7-MRP4-2 and MCF7-MRP4-3) along with a control line stably expressing empty vector (MCF7-Vec). MRP4 expression in MCF7-MRP4-2 and MCF7-MRP4-3 cells was 70-100-fold higher than in MCF7-Vec cells (Figure 4A). The ectopically expressed MRP4 protein was determined to be the correct size via western blot suggesting that post-translational modifications, such as glycosylation, were properly applied. Compared to MDA-MB-436 cells, MCF7-Vec cells accumulate less PGE_2_ overall, reflecting the lower endogenous COX-2 levels observed in these cells (Figure 1 and Figure 4). PGE_2_ release is modestly increased by stimulation with lipopolysaccharide (LPS, 10 ug/mL, 24 hours), but this increase was not statistically significant and was not further enhanced by over-expression of MRP4 in these cells (Figure 4B). This suggests that the combination of COX-2 expression and enforced MRP4 expression in MCF7 cells is not sufficient to accumulate PGE_2_ in the conditioned media.

In order to confirm that these genetic (RNA-interference and over-expression) and pharmacologic approaches were specifically affecting MRP4 functions, we employed a drug resistance assay to determine the effect of these perturbations on the sensitivity of these cells to the cytotoxic drug 6-mercaptopurine (6-MP), a known substrate for MRP4 [45]. In the instance of high MRP4 expression and activity, 6-MP does not accumulate in the cell to the degree that causes cytotoxicity and apoptosis; this reduced cytotoxicity is expressed as an increase in IC50.

MRP4 expression was suppressed via siRNA or shRNA in MDA-MB-231, MDA-MB-436, and BT549 cells, and sensitivity to 6-MP was determined and expressed as the IC50. The ratio of the IC50 of cells transfected with siRNA against ABCC4 compared to the IC50 of control transfected cells was calculated and this ratio is expressed as “fold sensitization.” In MDA-MB-231 cells (moderate basal MRP4 expression), reducing the level of MRP4 by either siRNA or shRNA resulted in an approximate 2-fold increased sensitivity to 6-MP (Table 1). Clones MDA-MB-231 shMRP4-B and MDA-MB-231 shMRP4-C had an approximate 80% decrease in MRP4 expression relative to vector control (data not shown). In MDA-MB-436 and BT549 cells (high basal MRP4 expression), reducing the expression of MRP4 using siRNA resulted in a 1.43–1.74-fold increase in cytotoxicity mediated by 6-MP. These data are consistent with a mechanism by which reduced MRP4 expression leads to reduced export of 6-MP resulting in higher accumulation of 6-MP and enhanced cell killing. Conversely, MCF7 cells that stably over-express MRP4 (MCF7-MRP4-2, MCF7-MRP4-3) were approximately 2-fold more resistant to 6-MP (higher IC50) when compared to MCF7 cells expressing empty vector, consistent with increased export of 6-MP (Table 1). Consistent with the genetic data, MRP4 inhibition with MK571 or Tyrphostin AG 1478 also increased the sensitivity to 6-MP in the three MRP4-expressing basal cell lines. Table 2 summarizes the fold sensitization as a result of treatment with MK571 or Tyrphostin AG 1478. Inhibitor concentrations were selected such that inhibitor treated cells had equivalent viability to vehicle-treated cells after 3 days. Pharmacologic inhibition of MRP4 by MK571 resulted in a 1.47, 1.76, and 2.94-fold increase in sensitivity to 6-MP in MDA-MB-436, BT549, and MDA-MB-231 cells, respectively. Tyrphostin AG 1478 (10 μM) induced a 2.86- and 4.83-fold increase in sensitivity to 6-MP in MDA-MB-436 and BT549 cells, respectively. The modulation of resistance to 6-MP by targeting MRP4 shows that these perturbations specifically affect MRP4 and not the entire PGE_2_ pathway.

**Table 1:** Genetic suppression of MRP4 increases sensitivity to 6-MP while ectopic over-expression of MRP4 increases resistance to 6-MP

Genetic Treatment	Fold Sensitization^a^		
**MDA-MB-231**			
siRNA	Construct A	2.25	**
	Construct B	2.22	*
shRNA	Clone 2	2.22	*
	Clone 4	1.97	*
**MDA-MB-436**			
siRNA	Construct A	1.43	*
	Construct C	1.63	*
**BT549**			
siRNA	Construct A	1.60	**
	Construct C	1.74	*
**MCF7**	**Fold Resistance^b^**		
MRP4 stable overexpression	Sub-line M2	1.93	**
	Sub-line M3	2.12	*

^a^Sensitization by RNA-interference relative to control transfection

^b^Resistance by MCF7 cells expressing MRP4 relative to vector control

* p < 0.05, ** p < 0.01

**Table 2:** Pharmacologic inhibition of MRP4 increases sensitivity to 6-MP

Pharmacologic Treatment	μM	Fold Sensitization^a^	
**MDA-MB-231**			
MK571	50	2.94	*
**MDA-MB-436**			
MK571	25	1.47	*
Tyrphostin AG 1478	10	2.86	**
**BT549**			
MK571	50	1.76	**
Tyrphostin AG 1478	10	4.83	**

^a^Sensitization by MRP4 inhibitors relative to vehicle treatment

* p < 0.05, ** p < 0.01

Independent of MRP4 expression level, treatment of MDA-MB-231 cells with a range of exogenous PGE_2_ did not significantly affect proliferation of these cells (data not shown). Additionally, we saw no significant change in proliferation when MRP4 protein levels were altered genetically (data not shown).

**Figure 5:**
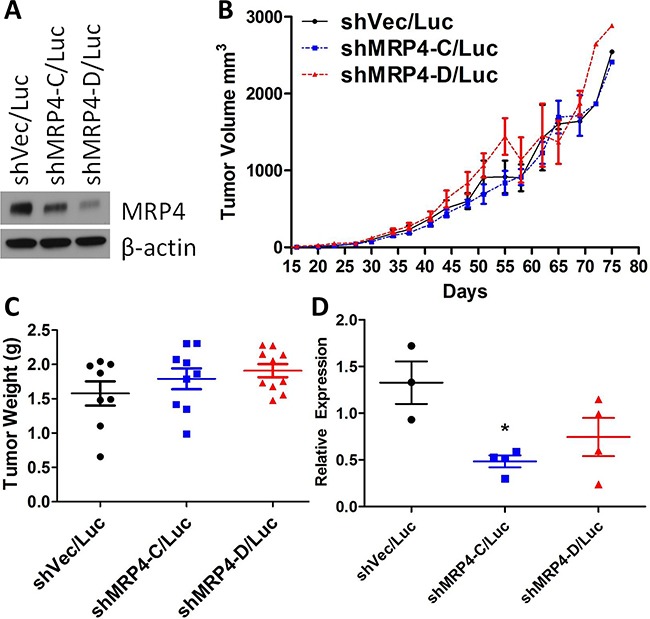
Subcutaneous xenografts of MDA-MB-231 shMRP4/Luc cells showed no difference in growth despite differences in MRP4 expression **A**. Western blot showing relative MRP4 expression levels of vector control (shVec/Luc) and shRNA knockdown clones (shMRP4-C/Luc and shMRP4-D/Luc) prior to injection in mice. β-actin was used as a loading control. **B**. Tumor volume (mean ± SEM) estimated from diameter measurements of subcutaneous tumors. Volume = (long diam.)(perpendicular diam.)^2^(π/6). **C**. Weight of excised subcutaneous MDA-MB-231 shRNA/Luc tumors. **D**. Relative expression of ABCC4 mRNA from excised MDA-MB-231 shMRP4/Luc xenograft tumors. Each point represents one tumor sample. * p = 0.0093 compared to MDA-MB-231 shVec/Luc tumor samples.

Since elevated COX-2 and PGE_2_ levels are indicators of poor prognosis in several types of cancer including breast, and we have shown that MRP4 contributes to the amount of PGE_2_ produced by breast cancer cells, we investigated the role of MRP4 on both primary tumor growth and metastatic potential. MDA-MB-231 cells (5×10^5^) stably expressing vector control or shRNA targeting MRP4 along with stable expression of the luciferase enzyme (MDA-MB-231 shRNA/Luc, Figure 5A) were implanted subcutaneously proximal to the mammary fat pad of female BALB/c SCID mice; tumor growth was monitored by caliper, and metastatic spread was estimated by bioluminescent imaging [46]. At day +90, 9 of 10 mice injected with either MDA-MB-231 shVec/Luc or MDA-MB-231 shMRP4-C/Luc had developed subcutaneous tumors. All ten mice injected with MDA-MB-231 shMRP4-D/Luc cells had established subcutaneous tumors. Mean tumor volumes were not different among mice injected with control versus either MRP4 knockdown cell line (Figure 5B). Upon reaching 18 mm in longest tumor diameter, mice were euthanized; blood, lung, and tumor tissues were collected for analysis. Tumor weight upon necropsy was used as a confirmatory measure of tumor growth. Consistent with the estimated tumor volumes, excised tumor weight was not significantly different among the three cell lines expressing different levels of MRP4 (Figure 5C). We observed a similar expression pattern of ABCC4 mRNA among the tumor tissues generated from the knockdown cell lines (Figure 5D) compared to the protein expression of the primary cell lines used for injection (Figure 5A). Tumors derived from MDA-MB-231 shVec/Luc cells had higher ABCC4 mRNA expression compared to tumors derived from MDA-MB-231 shMRP4-C/Luc or MDA-MB-231 shMRP4-D/Luc MRP4 knockdown clones indicating that the tumor MRP4 phenotype at the end of the observation period reflected the phenotype of the initial implanted tumor cells. Based on these observations, we conclude that the absence of any effect of MRP4 downregulation on primary tumor growth is not due to upregulation of MRP4 *in vivo*.

**Figure 6:**
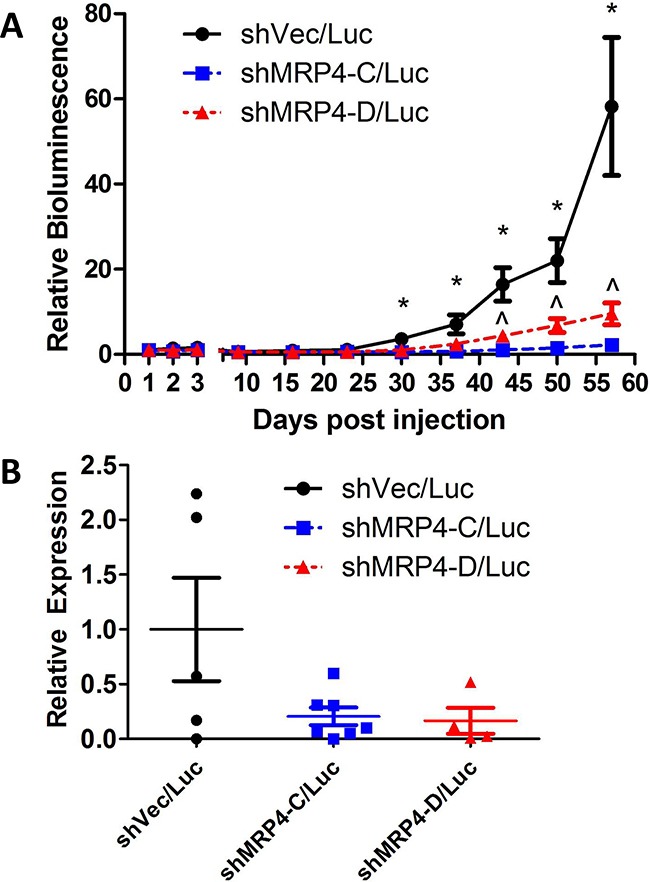
Suppressed expression of MRP4 decreased metastatic load in mice bearing subcutaneous MDA-MB-231 shMRP4/Luc tumors Weekly pulmonary region bioluminescence readings were taken from 9-10 mice/group with established subcutaneous MDA-MB-231 shMRP4/Luc tumors. **A**. Bioluminescence signal was corrected for background luminescence and mean ± SEM of 9-10 mice per group is reported relative to 1 day post-injection pulmonary bioluminescence. The following symbols represent the results from t-tests performed at each timepoint. *: p < 0.05 between vector and knockdown clones (shVec/Luc vs. shMRP4-C/Luc, shVec/Luc vs. shMRP4-D/Luc), ^: p < 0.05 between knockdown clones (shMRP4-C/Luc vs. shMRP4-D/Luc). **B**. Relative expression of human GAPDH relative to mouse Gapdh in lung samples from mice that bore the indicated tumor xenografts.

Whole-animal bioluminescence imaging was monitored over the course of tumor growth in order to detect spontaneous metastases from the mammary gland-implanted tumor. We detected markedly more bioluminescence in the lungs of mice injected with vector control MDA-MB-231 shVec/Luc cells compared to the mice injected with either of two MRP4 knockdown clones (MDA-MB-231 shMRP4-C/Luc and MDA-MB-231 shMRP4-D/Luc) (Figure 6A). Thus, differences in MRP4 expression did not impact the growth of the primary tumor, but had a profound effect on the ability of tumor cells to establish spontaneous pulmonary metastases. Bioluminescence in the lungs of mice injected with shVec/Luc cells increased over time, consistent with the continuing expansion of metastatic lesions whereas very little, if any, growth of either cell line expressing shMRP4 occurred during the observation period. The reduced metastatic potential in MRP4 knockdown cells was confirmed in a second independent experiment (data not shown). In order to confirm the presence of human breast cancer cells in the lung tissue of these mice, total RNA was isolated from lung tissue and evaluated by qPCR for the presence of human GAPDH (using human-specific GAPDH primer set) compared to mouse Gapdh mRNA. When the average relative expression of GAPDH/Gapdh detected in the MDA-MB-231 shVec/Luc samples was set equal to 1, lower levels of GAPDH/Gapdh were observed in the lungs of mice bearing tumors with suppressed MRP4 expression (MDA-MB-231 shMRP4-C/Luc, MDA-MB-231 shMRP4-D/Luc) (Figure 6B).

## DISCUSSION

We recently reported the analysis of gene expression of COX-2 pathway members in a large publicly available breast cancer database [39]. That study identified a pattern of expression in TNBC and basal type breast cancer which should result in high PGE_2_ in the tumor microenvironment i.e., high COX-2, high MRP4, low PGT, and low 15-PGDH. No biochemical data was available in that dataset which prompted the current study to characterize the functional importance of this pattern. The current study further supports our central hypothesis that the COX-2/PGE_2_ pathway is dysregulated in aggressive cancers [7, 8, 47]. We have now shown that breast cancer subtypes have different expression profiles of the PGE_2_ pathway members and that MRP4 expression was strongly elevated in aggressive (IDC, TNBC, basal type) breast cancer cell lines relative to less aggressive, luminal cell lines. Given that MRP4 is also expressed in normal epithelium, it was not surprising that we detected this protein in MCF10A cells; however, in malignant cell lines, MRP4 expression correlated with an aggressive phenotype [13, 40]. We also saw a trend of decreased 15-PGDH protein expression in aggressive breast cancer cell lines relative to normal mammary epithelium consistent with the reported tumor suppressor role of 15-PGDH [35, 37]. These data support the hypothesis that other members of the PGE_2_ pathway besides COX-2, in particular MRP4, PGT, and 15-PGDH, may have an impact on the intratumoral levels of PGE_2_ [5, 7, 9, 34, 38, 48].

Previously unknown, we have now shown that breast cancer cells with elevated MRP4 expression would be able to more efficiently export PGE_2_ into the microenvironment. A tumor microenvironment with elevated PGE_2_ could be established and sustained when MRP4 expression is elevated even in tumors in which COX-2 expression is not elevated, and this identifies a second potential mechanism, beyond COX-2, to achieve high PGE_2_ in the tumor microenvironment. This model of high-MRP4 expression also assumes low expression of PGT and 15-PGDH which would otherwise metabolize intratumoral PGE_2_. We demonstrate that, in TNBC particularly, MRP4 could also play a role in maintaining a tumor microenvironment with elevated PGE_2_ levels, a clinical parameter that has been associated with poor outcome [5, 15, 49, 50]. Notably, MRP4 but not MRP1-3 or MRP5 transports PGE_2_ (and PGE_1_) [51]. In addition to PGE_2_, MRP4 exports nucleoside analogs, e.g. cAMP and cGMP, which could contribute to malignant behavior. While previous findings from our group and others suggest that metastatic potential downstream of PGE_2_/EP4 signaling is not strictly dependent on cAMP [16, 19, 52], we cannot rule out a role of nucleoside transport by MRP4. Future studies will examine these additional potential mechanisms by which MRP4 promotes metastasis.

We have now shown that suppressing MRP4 expression leads to decreased metastatic potential. We showed previously that PGE_2_ activates EP4 to support breast cancer metastasis [16, 53]. Our current studies support the hypothesis that elevated MRP4 could be a mechanism underlying high PGE_2_/EP4 signaling and this mechanism may be particularly important in basal and TNBC. There is extensive literature demonstrating that high PGE_2_ is associated with enhanced metastatic potential [5, 47, 54]. Consistent with this mechanism, we observed reduced metastatic capacity from mammary gland implanted tumors with reduced MRP4 expression. Growth of primary tumors was not apparently affected by changes in MRP4 levels.

PGE_2_ elicits diverse biological responses in tumor cells and heterogeneous tumor tissue in a cell type-specific manner [42]. PGE_2_ signaling is not directly associated with proliferation of MDA-MB-231 breast tumor cells. Increased MRP4 expression has been correlated with increased proliferation in other tumor types but this was not linked mechanistically to PGE_2_ production [24, 27, 28, 31]. Reducing expression of MRP4 in either MDA-MB-436 or MDA-MB-231 cells did not affect cell proliferation *in vitro*. We have shown that MRP4 contributes to PGE_2_ accumulation *in vitro* and metastatic progression *in vivo*, but this does not definitively exclude the possibility that another substrate of MRP4 besides PGE_2_ is also contributing to these effects. MDA-MB-436 cells express both COX-1 and COX-2, and PGE_2_ content in the conditioned media of these cells is influenced by the activity of MRP4. Consistent with the proliferation data, we did not see differences in primary tumor growth with respect to MRP4 expression level in MDA-MB-231 xenograft experiments; we further conclude that MRP4 does not directly affect cell proliferation. These data are consistent with the reports that PGE_2_ is not directly associated with proliferation in all breast cancer [42].

Taken together, we show that suppression of MRP4 expression in a metastatic, basal type breast cancer cell line (MDA-MB-231) decreased the ability of a subcutaneous primary tumor to develop spontaneous metastases when compared to MDA-MB-231 cells with high endogenous levels of MRP4. Our findings are consistent with our central hypothesis that MRP4 is functioning in the tumor microenvironment of the established primary tumor by increasing the level of PGE_2_ which can act in an autocrine or paracrine manner and is available to diverse cells in the heterogeneous tumor, enhancing metastatic potential and progression of the tumor. MRP4 could be a contributing factor in the accumulation of PGE_2_ in the tumor microenvironment preferentially in TNBC and basal subtype tumors compared to luminal breast tumors and should be considered as a potential molecular target in this subtype with overall poor outcomes.

## MATERIALS AND METHODS

Human cell lines originally obtained from ATCC (Manassas, VA) were cultured in a 5% CO_2_ atmosphere at 37°C. MCF10A cells were maintained in DMEM/F12 (Corning, Corning, NY) supplemented with 5% horse serum, 20 ng/ml epidermal growth factor, 10 μg/ml insulin, 1 ng/ml cholera toxin, 100 μg/ml hydrocortisone, 1% penicillin and 1% streptomycin. The following breast cancer cell line media (Corning) was supplemented with 10% fetal bovine serum (FBS) (Gemini Bio-products, Atlanta Biologicals), 1% penicillin/streptomycin (Gemini Bio-products), and 2 mM L-glutamine (Gemini Bio-products). MCF7, MDA-MB-231, and MDA-MB-468 cells were grown in Dulbecco's Modified Eagle Medium (DMEM) with 1 g/L glucose. T47D cells were grown in RPMI 1640 medium. MDA-MB-436 cells were grown in DMEM/F12 medium. BT549 cells were grown in DMEM with 4.5 g/L glucose. Cell lines were recently (June, 2016) authenticated by STR typing using the Promega Geneprint 10 system in comparison to ATCC STR databases. At the same time, the GenePrint 5x mouse primer pair mix was used to rule out contamination with mouse DNA.

Control and shRNA plasmids (Origene) targeting ABCC4 were transfected into retroviral packaging phoenix cells (Allele Biotechnology, San Diego, CA) with Lipofectamine 2000 (Thermo Fisher). Forty-eight hours after transfection, retrovirus-containing medium was collected and centrifuged to pellet any cell debris. The supernatant containing the viral particles was transferred to new tubes and stored at -80°C. Viral medium was added with 4 μg/mL polybrene (American Bioanalytical) to MDA-MB-231 cells for 2 days before being passaged into 1.0 μg/mL puromycin (Invitrogen) selection. After 1 week of selection, surviving cells were characterized by quantitative PCR and western blot for ABCC4/MRP4 knockdown compared to vector control cells, and the pooled populations with the greatest knockdown were subcloned for further characterization. Clones derived from puromycin-resistant single-cell cultures with stable MRP4 knockdown were cultured with viral media containing a luciferase expression plasmid for 3 days before being passaged into hygromycin B selection (Corning). After 2 weeks, resistant cells were characterized for luciferase expression by adding luciferin (PerkinElmer) (250 μg/mL) to the cells and evaluating bioluminescent intensity via luminometer (Berthold Technologies, U.S.A., Oak Ridge, TN). Signal 200-fold over background was considered sufficient for use in *in vivo* experiments.

MRP4 over-expression was performed both transiently and stably. Transient transfection of MCF7 cells with the MRP4 expression plasmid pcDNA3.1(-)MRP4-Zeo (a generous gift from H. Hayashi, University of Tokyo) or pcDNA3.1(+)-Zeo (a generous gift from I. Lindberg, University of Maryland) empty vector was conducted using Lipofectamine 3000 (Thermo Fisher) at a ratio of 2 μg DNA to 3 μL Lipofectamine 3000. Stable MRP4 expressing MCF7 sub-lines were generated similar to the transient cells, but with the addition of Zeocin (100 μg/mL) (Thermo Fisher) to growth media. Cells were passaged every 3-4 days with fresh Zeocin. After 3 weeks, surviving cells were characterized by western blot for relative MRP4 expression. Two cell lines expressing MRP4 (MCF7-MRP4-2 and MCF7-MRP4-3) and one cell line expressing vector (MCF7-Vec) were used to evaluate PGE_2_ accumulation and 6-MP resistance.

### RNA, cDNA, qPCR

Total RNA was isolated from cultured cells using the NucleoSpin RNA kit (Machery-Nagel) according to the manufacturer's instructions. Isolation of RNA for siRNA screening was performed using the DirectZol RNA isolation kit (Zymo). Total RNA from mouse tissue was isolated using TRIzol following the manufacturer's protocol (Thermo Fisher Scientific). cDNA was synthesized from 500-1000 ng total RNA using the qScript cDNA SuperMix (Quanta) according to the manufacturer's instructions. ABCC4 and GAPDH expression were performed in triplicate using probe-based primer sets and iQ Supermix (Bio-Rad) with approximately 100 ng cDNA per reaction. Relative gene expression was determined using the 2^-ΔΔCt^ method with GAPDH as the reference gene. Results are representative of replicate experiments and expressed as relative expression ± standard deviation [55].

### Protein isolation

Total cellular protein was collected from cultured cells following a wash with cold phosphate buffered saline (PBS). Lysis buffer was comprised of RIPA buffer (Sigma-Aldrich) supplemented with 1% protease inhibitor (Sigma-Aldrich), 1% phenylmethylsulfonyl fluoride (PMSF, Sigma-Aldrich), sodium orthovanadate (2 mM, Sigma-Aldrich), and sodium fluoride (5 mM, New England Biolabs). Lysis buffer was added to adherent cells and incubated on ice for 10 minutes. Alternatively, cells were detached using trypsin, resuspended in growth media, and centrifuged. The cell pellet was resuspended in lysis buffer. Lysates were vortexed 2-3 times over 20 minutes and otherwise kept on ice. Lysates were clarified by centrifugation at 8000 x g for 10 minutes at 4°C. Clarified, soluble protein was transferred to a new tube and stored at -80°C. Protein concentration of these clarified lysates was determined by the Bradford protein quantification assay (Thermo Fisher Scientific).

### Western immunoblotting

Equal amounts of protein (20-50 μg) were combined with 4x Laemmli sample buffer and β-mercaptoethanol (2.5% final) (Bio-Rad) and incubated at 95°C for 5 minutes before being loaded into SDS-PAGE gels for electrophoresis. Separated proteins were transferred to PVDF membrane using the Trans-Blot Turbo system (Bio-Rad) and blocked in 5% milk in wash buffer (phosphate buffered saline plus 0.1% Tween-20, PBS-T). Membranes were incubated overnight at 4°C with primary antibodies under gentle rocking. Membranes were washed and incubated for 1 hour with secondary antibodies at room temperature under gentle rocking. Membranes were incubated in an ECL (Pierce, Bio-Rad, or GE) reagent for 5 minutes and exposed to x-ray film to obtain the relative protein expression. Primary antibodies against COX-2, EP4, 15-PGDH, and PGT were from Cayman Chemical (Ann Arbor, MI). Primary antibodies against COX-2 and COX-1 were from Cell Signaling Technologies (CST). Primary antibody against MRP4 was from Enzo Life Sciences (M4I-10). The primary antibody against beta-actin (AC-15) was from Sigma-Aldrich. Milk (5%) in PBS-T was used for diluting primary and secondary antibodies. Horseradish Peroxidase (HRP)-conjugated secondary antibodies were used in the following concentration ranges: anti-rabbit (Bio-Rad) 1:5,000, anti-mouse (KPL) 1:5,000 – 1:10,000, and anti-rat (CST) 1:3,000 – 1:5,000.

### PGE_2_ determination assay

PGE_2_ levels in conditioned media were determined using the Prostaglandin E_2_ EIA kit (Cayman Chemical) according to the manufacturer's protocol. For normalization, PGE_2_ content is expressed in pg/μg cellular protein. When using MDA-MB-231 cells for PGE_2_ export experiments, cells were stimulated with 80 nM PMA (Sigma-Aldrich) in fresh growth medium for 1 hour at 37°C. Stimulation media was removed and replaced with fresh growth media for 16 hours at 37°C. The conditioned medium and whole cell lysate were collected and stored at -80°C. MRP4 inhibitors were added to both stimulation and replacement media when indicated. MCF7 cells were stimulated with 10 μg/mL lipopolysaccharide (LPS, Sigma-Aldrich) for 24 hours and conditioned medium was collected along with total cellular protein.

### Chemicals

MK571 (Cayman, Calbiochem) was dissolved to 15 mM in DMSO, aliquoted, and stored at -20°C. Tyrphostin AG 1478 (Sigma-Aldrich) was dissolved to 10 mM in DMSO and stored at 4°C. 6-Mercaptopurine (6-MP, Sigma-Aldrich) was dissolved to 250 mM in 1 M NaOH (Sigma-Aldrich) and stored at -20°C for up to 2 weeks. A 4-fold dilution series of 6-MP was prepared in 1 M NaOH and added to growth media so that the maximal final NaOH concentration was 4 mM. There were no proliferative changes between cells cultured in growth media alone or in growth media with 4 mM NaOH. Luciferin (PerkinElmer) was dissolved in sterile PBS to 40 mg/mL and stored, protected from light, at -20°C.

### Human breast cancer xenograft

MDA-MB-231 cells (5×10^5^) stably expressing both shRNA targeting ABCC4 and luciferase were injected subcutaneously into female BALB/c-SCID mice (Jackson). The MDA-MB-231 cell line was selected for these xenograft experiments as it reliably established tumors and spontaneous metastatic lesions. Tumor growth was measured twice weekly and approximate tumor volume was calculated by “Volume = (A)(B^2^)(π/6),” where A = long diameter and B = perpendicular diameter. All animal experiments were conducted in compliance with University of Maryland Institutional Animal Care and Use Committee.

### Statistical analysis

The Student's t-test was used to compare two experimental groups. A p-value < 0.05 was considered to be significant (*). 1-way ANOVA was used to analyze multiple experimental conditions, and a Bonferroni post-test was used to determine the significance of each pair of conditions tested. Results are representative of at least 3 experiments.
